# Toxic Megacolon Burdened with COVID-19 Coinfection—Worsening of an Unfavorable Diagnosis: A Single-Center Retrospective Study

**DOI:** 10.3390/life12101545

**Published:** 2022-10-05

**Authors:** Tomáš Řezáč, Dušan Klos, Martin Stašek, Radek Vrba, Pavel Zbořil, Petr Špička

**Affiliations:** 1 Department of Surgery I, University Hospital Olomouc, I.P. Pavlova 185/6, 77900 Olomouc, Czech Republic; 2 Department of Surgery I, Faculty of Medicine and Dentistry, Palacky University Olomouc, Hněvotínská 976/3, 77515 Olomouc, Czech Republic

**Keywords:** toxic megacolon, Clostridium difficile infection, COVID-19 infection, Candida species, Klebsiella species, prognosis

## Abstract

Introduction: This study primarily sought to evaluate the risk factors for toxic megacolon development and treatment outcomes in Clostridium difficile-positive COVID-19 patients, secondarily to determining predictors of survival. Methods: During the second COVID-19 wave (May 2020 to May 2021), we identified 645 patients with confirmed COVID-19 infection, including 160 patients with a severe course in the intensive care unit. We selected patients with Clostridium difficile infection (CDI) (31 patients) and patients with toxic megacolon (9 patients) and analyzed possible risk factors. Results: Patients who developed toxic megacolon had a higher incidence (without statistical significance, due to small sample size) of cancer and chronic obstructive pulmonary disease, a higher proportion of them required antibiotic treatment using cephalosporins or penicillins, and there was a higher rate of extracorporeal circulation usage. C-reactive protein (CRP) and interleukin-6 values showed significant differences between the groups (CRP [median 126 mg/L in the non-toxic megacolon cohort and 237 mg/L in the toxic megacolon cohort; *p* = 0.037] and interleukin-6 [median 252 ng/L in the group without toxic megacolon and 1127 ng/L in those with toxic megacolon; *p* = 0.016]). As possible predictors of survival, age, presence of chronic venous insufficiency, cardiac disease, mechanical ventilation, and infection with Candida species were significant for increasing the risk of death, while corticosteroid and cephalosporin treatment and current Klebsiella infection decreased this risk. Conclusions: More than ever, the COVID-19 pandemic required strong up-to-date treatment recommendations to decrease the rate of serious in-hospital complications. Further studies are required to evaluate the interplay between COVID-19 and CDI/toxic megacolon.

## 1. Introduction

### 1.1. Background

Coronavirus disease 2019 (COVID-19), caused by severe acute respiratory syndrome coronavirus 2, has caused the biggest health emergency in recent decades [1,2]. Since 31 December 2019 when the World Health Organization (WHO) reported a new respiratory disease that was affecting the Chinese city of Wuhan, the virus has spread to all continents and is responsible for over 30 million infections and 950,000 deaths [3]. 

COVID-19 was a new disease without clear recommendations but with significant differences in treatment due to local practices or recommendations. Given the significant bacterial superinfection that occurs with COVID-19, and in order to protect other patients and healthcare professionals as much as possible, one of the first recommendations, although the disease is viral, was the prophylactic use of combination antibiotic therapy. Up to 94% of COVID-19 patients have been reported to receive empirical antibiotic therapy during their hospital stay [4,5].

However, in recent times, cases of co-infection with Clostridium difficile in patients with COVID-19 and, quite rarely, the development of toxic megacolon have been reported. The development of Clostridium difficile (CDI) and Candida infection is considered to be associated with the use of broad-spectrum antibiotics, inadequate infection control, and the overcrowding of hospital beds, which is aggravated by the critical COVID-19 situation [6]. The progression of CDI to symptomatic CDI remains a complex and challenging problem. A possible explanation is intestinal damage due to severe acute respiratory syndrome coronavirus 2 infection. It can extensively attack all organs that express angiotensin-convertase enzyme 2 (ACE2), the angiotensin-convertase enzyme 2 (toxic megacolonPRSS2) receptor, and the cellular serine protease—transmembrane serine protease 2: gastrointestinal tract, heart, kidney, and vast distal vasculature [7]. In addition, several pieces of evidence suggest that gut microbiota disequilibrium is necessary for Clostridium difficile colonization [8,9].

### 1.2. Objectives

This retrospective study aimed to assess the risk factors for toxic megacolon development and treatment outcomes in Clostridium difficile-positive COVID-19 patients, to evaluate the outcomes of treatment for this disease, and to outline prevention and treatment options and secondarily to determine predictors of survival.

## 2. Results

### 2.1. Participants

Thirty-one patients met the criteria for a positive COVID-19 diagnosis, intensive care unit (ICU) admission, and confirmed clostridial infection. Nine of these patients developed toxic megacolon.

### 2.2. Descriptive Data

The median age in the cohort was 65 years, with a maximum of 88; higher age was predominant in the toxic megacolon group; the median BMI was 30.7 kg/m^2^, with a maximum of 52.6 kg/m^2^, and higher BMI was predominant in the group without TM (*p* = 0.233); the median number of days requiring ICU admission was 8, with a slightly lower median in the group with TM (*p* = 0.524), indicative of severe and rapid disease development. Further examination of basic demographic data did not reveal statistically significant relationships between these parameters and the occurrence of toxic megacolon. Only a current medical history of cancer and COPD were slightly higher in the group that developed toxic megacolon, but the results did not reach statistical significance. All patients were treated with corticosteroids, with no proven statistical significance. Regarding antibiotic therapy before and during ICU admission, only treatment with cephalosporins and penicillins resulted in the development of toxic megacolon, but the results were not statistically significant (Table 1).

### 2.3. Main Results

Of laboratory values, only the CRP level at admission reached statistical significance (median, 126 mg/L) in the toxic megacolon cohort (237 mg/L, *p* = 0.037), Table 2.

Of the maximum inflammation values, interleukin 6 was statistically significant for toxic megacolon development (median 422 ng/L) in the group without toxic megacolon (252 ng/L) and those with toxic megacolon (1127 ng/L); the maximum was 8842 ng/L, *p* = 0.016.

Additionally, 35.5% of the patients had self-infection at the time of admission; the acquired nosocomial infections included mostly Pseudomonas, 29% (Aeruginosa 7×, Mosseli 1×); Enterococcus faecium, 25.8% (vancomycin-resistant 2×); Burkholderia multivorans, 25.8%; Candida sp., 19.4% (Albicans 4× and Tropicalis and Dubliniensis both 1×); Serratia marcescens, 19.4%; other infections were less predominant, and none of them influenced toxic megacolon development (Staphylococcus haemolyticus, 16.1%; Enterobacteriaceae, 9.7% (Cloacae 2×, Faecium 1×); Klebsiella variicola, ESBL + 9.7%; other rare strains in Table 3). We considered nosocomial infection to be any bacterial strain that was cultured during hospitalization and was different from the primary, initial bacterial culture.

Using the Glasgow coma scale, the initial values for all patients were 4, 5, and 6, without statistical significance. The median clinical frailty score in both patients with toxic megacolon and in those without toxic megacolon was 3, and the median initial ATLAS score in both groups was 4, with no statistically significant difference between the groups. For the ATLAS score, mortality outcomes were significantly affected by a current history of COVID-19 (ATLAS 2 33.3% vs. 0.0% in those without COVID-19 co-incidence, and ATLAS 3 25.0–28.6% vs. 3.6%). ATLAS 4 42.9–74.7% vs. 4.2% and an ATLAS score of 5 or more shows a 100% risk vs. 8.7% (score 5), 10.9% (score 6), 14.3% (score 7), and 56.0% (score 8 or more) in non-COVID 19 patients [10].

Operative revisions for toxic megacolon were performed twice, with ATLAS scores of 5 and 6, but both revisions resulted in the death of the patient. In those with an overall good clinical condition, such as those who have survived until the time of this publication, 2-time conservative management was performed, and the bowel was endoscopically desufflated. In the remaining five patients, conservative management was also applied, due to the infaust prognosis of the patients and the likelihood of the management not influencing infaust prognosis. 

The difference in survival (33 days, which was the longest hospitalization ending in death) between patients with toxic megacolon and those with clostridial colitis and COVID-19 infection did not reach statistical ignificance (*p* = 0.791). The remaining patients survived without significant complications.

Finally, we evaluated all parameters as possible predictors of survival and assessed parameters that increased and decreased the risk of death. We evaluated age, presence of chronic venous insufficiency, cardiac disease, mechanical ventilation, and the presence of Candida species as significant parameters that increased the risk of death, while significant parameters that decreased the risk of death included corticosteroid treatment, antibiotic prophylaxis using cephalosporins, and current Klebsiella infection (Table 4).

## 3. Discussion

COVID-19 and Clostridium difficile infection (CDI-19) are rarely reported together; this association has been linked to severe complications [11]. Diarrhea is one of the most common non-pulmonary symptoms in patients with COVID-19 [12], and 10% of patients additionally present with abdominal pain and vomiting. According to some studies, this incidence was as high as 19.4% [13,14], and this can act as a confounding factor and delay diagnosis and CDI treatment, leading to poor outcomes. In our series of 160 patients in the ICU, no patient experienced abdominal discomfort or diarrhea in the prehospital period. The underlying problems were dyspnea, diarrhea developed only during hospitalization due to rather iatrogenic reasons, parenteral nutrition, corticosteroid treatment, nosocomial infections, and the combination of antibiotics. When diarrhea was present, a stool sample was sent to the laboratory to assess for CDI; this examination was often repeated during hospitalization to avoid delaying the diagnosis. Subsequently, treatment comprising oral glycopeptide antibiotic therapy (vancomycin) or vancomycin in combination with metronidazole at an intravenous dose of 500 mg 3 times daily was administered (recommendation for nonfulminant episodes).

Clostridium difficile is a Gram-positive, spore-forming, anaerobic bacillus that is widely distributed in the intestinal tract of humans, animals, and the environment. Approximately 5% of adults and 15–70% of infants are infected with Clostridium difficile, and the prevalence is several times higher in hospitalized patients [15] and among patients in ICU (up to 51%) [16]; in our case, the prevalence was less than 20%. 

Significant patient-related risk factors for CDI include antibiotic exposure, older age, and hospitalization. The clinical symptoms range from mild, self-limiting diarrhea to fulminant colitis, and CDI can include pseudomembranous colitis, toxic megacolon, bowel perforation, and sepsis or multiple organ dysfunction syndrome [17]. 

Antibiotic use has long been associated with increased risks of CDI [odds ratio (OR) 3.55, 2.56–4.94]. Brown et al. found that clindamycin [OR = 16.80, 95% confidence interval (CI) 7.48–37.76], fluoroquinolones (OR = 5.50, CI 4.26–7.11), and carbapenems (OR = 5.68, CI 2.12–15.23) were the most likely antibiotics to be associated with CDI, while macrolides (2.65, CI 1.92–3.64) and penicillins (OR = 2.71, CI 1.75–4.21), although problematic, were less so [18].

In our series, the development of toxic megacolon was associated with antibiotic treatment using penicillins and cephalosporins, but the results did not reach statistical significance due to the small sample size, which was the main limitation of the study and the cause of bias. Female sex, age > 40 years, hypoalbuminemia, acidosis, and high blood urea nitrogen levels are associated with high mortality. In our study, older age was also confirmed to be a risk factor (*p* = 0.042, RR = 1.034, CI = 1.001–1.069), as well as the presence of chronic venous insufficiency (*p* = 0.021, RR = 7.445, CI = 1.352–41.0), cardiac disease (*p* = 0.021, RR = 3.220, CI = 1.194–8.680), mechanical ventilation (*p* = 0.045, RR = 7.909, CI = 1.044–59.9), or colonization with Candida albicans (*p* = 0.039, RR = 3.091, CI = 1.059–9.024). Hypoalbuminemia was not present as a risk factor, even in the report; patients who developed toxic megacolon had a higher median compared to patients without TM (34 g/L vs. 32.1 g/L). The situation was analogous in the evaluation of renal function: the group with TM had better creatinine values than the group without TM (77 µmol/L vs. 87 µmol/L).

The main objective of all medical therapy is to avoid the need for surgical management; antibiotics should be discontinued immediately in patients with toxic megacolon due to severe CDI colitis. Steroids are contraindicated in toxic megacolon due to an infectious etiology, including CDI colitis. In our study, steroids were administered as standard treatment in all patients with COVID-19, and according to our results, the steroid treatment significantly reduced the risk of exitus. 

Surgery is indicated in patients with colonic perforation, necrosis, full-thickness ischemia, intra-abdominal hypertension or abdominal compartment syndrome, clinical signs of peritonitis, worsening abdominal examination findings despite adequate medical therapy, or end-organ failure and high serum lactate level, when all other conservative treatment options have failed. 

The timing of surgery remains controversial, and delay may ultimately increase the risk of complications, such as abdominal compartment syndrome or perforation. Careful monitoring for any signs of impending perforation is vital. Intervention before colonic perforation has a lower mortality rate compared to colectomy after perforation (8% vs. 40%) [19]. 

In older studies, the overall mortality from toxic megacolon secondary to severe CDI was 64 to 67%, and it was 71 to 100% in patients that were managed surgically. It can be said that COVID-19 has set us back several decades in patient mortality, with an overall mortality of 66.6% (6/9 patients) and a 100% rate in patients who were managed surgically (2/2 patients). These studies show that patients will recover better with medical management alone and that surgery may only offer a nominal benefit. However, surgery should not be postponed in critically ill patients [20,21]

Colonoscopic decompression is safe and effective as a non-surgical treatment for toxic megacolon in approximately 57–71% of cases [22] and was also successful in our set-up. Curative treatment was achieved in 2/2 patients. We also found other treatment options, such as leukocytapheresis, hyperbaric oxygen therapy, or tacrolimus application, in previous case reports, but further studies are needed to confirm the results.

Patients with CDI-19 had an overall worse outcome (including a longer hospital stay [35 days versus 19.4 days, *p* < 0.01] and lower rates of full recovery without complications [50% versus 64.9%, *p* = 0.01]) than those without CDI [23]. 

Unfavorable results are potentiated by parameters that increase the risk of death, without the possibility of being influenced, these include age, presence of chronic venous insufficiency, cardiac disease, mechanical ventilation, and colonization with Candida albicans. Incidentally, to our knowledge, the role of yeast has not yet been described in the currently available literature. The progression to symptomatic infection is same as in CDI, the disruption of the skin or gastrointestinal barrier. The depletion of the immune system or changes in the balance of the microbiota, along with other factors, can facilitate the spread of candida. Unfortunately, prevention is problematic even in the non-COVID-19 period. Despite the availability of antifungal agents, disseminated candidiasis is accompanied by high mortality (about 40–60%), poor diagnosis, and inappropriate disease management. Antifungal resistance is a major problem and has even been reported in individuals who have not been exposed to antibiotics.

## 4. Methods

A stool sample was collected for culture in cases of diarrhea and suspected clostridial infection. CDI diagnosis was confirmed by the presence of toxins in the stool. Clinically, clostridial colitis was diagnosed if a patient had diarrhea and a positive stool culture without other gastrointestinal or general symptoms, and toxic megacolon due to CDI was diagnosed if the patient’s presentation met the diagnostic criteria, including

1. Radiological diagnosis of colonic dilatation of at least 6 cm;
2. At least one of the following criteria: temperature (>38.6 °C, 101.5 °F), tachycardia (>120 beats/min), leukocytosis (>10.5 × 103/μL), or anemia;
3. Any of the following: hypotension, hypovolemia, altered mental status, or electrolyte disorder.

A polymerase chain reaction test determined the diagnosis of COVID-19.

### 4.1. Study Design

We focused on patients with confirmed clostridial infection, patients who developed toxic megacolon, and treatment outcomes and to determine whether there are associations between individual risk factors

Setting: Study participants were recruited from the institutional database. Inclusion criteria were laboratory-confirmed COVID-19 infection, severe course with intensive-care-unit hospitalization, CDI positivity, and the development of toxic megacolon during the second COVID-19 wave, i.e., from May 2020 to May 2021

### 4.2. Participants

We identified 645 patients (347 males and 298 females) and 160 patients (100 males and 60 females) who had a severe course of the disease and were admitted to intensive care units. A total of 31 patients had confirmed clostridial infection, and 9 patients developed toxic megacolon.

Variables: primary demographic data, data on associated diseases and clinical status, treatment (corticosteroid, ATB before and after ICU admission), laboratory values of nutritional status, liver function, inflammation, blood count, coagulation, and essential ions, influence of primary/self-infection, and nosocomial infection acquired during ICU hospitalization.

Data source: Data were obtained from the institutional database.

Bias: The small group of patients, due to the specificity of the disease and comorbidities, limits the statistical significance of the cohort. However, for the same reason, it does not allow generalizable conclusions.

Statistical methods: IBM SPSS Statistics version 23 (IBM Corp., Armonk, NY, USA) was used for data analysis. For quantitative parameters, patients were compared using the Mann–Whitney U test. The Chi-square test and Fisher’s exact test were used to compare qualitative parameters. Kaplan–Meier analysis with log-rank test was used for survival analysis. Significant factors were determined using Cox regression analysis. Logistic regression analysis was used to calculate the odds ratio (OR) of each parameter. Normally distributed data were tested using the Shapiro–Wilk test. All tests were performed at a significance level of 0.05.

## 5. Conclusions

In conclusion, it is necessary to consider the risk of infections during any pandemic with increased hospitalization rates, antibiotic overuse, and difficulty maintaining hygiene. More than ever, pandemics require clear and up-to-date treatment recommendations to reduce the rate of severe hospital complications, which is sometimes very complicated at the beginning of a disease with strange behavior. From our limited study, a way to improve survival of a COVID-19- and, at the same time CDI-, positive patient is treatment with corticosteroids and cephalosporins, a conservative approach that is maximally recommended if the condition allows it. However, further studies involving more patients are needed to evaluate the interplay between COVID-19 and CDI and the risk factors for the development of toxic megacolon.

## Figures and Tables

**Table 1:** Characteristics of the study, basic demographic data, data on associated diseases and clinical status, treatment (corticosteroid, ATB before and after ICU admission), and its relation to CDI, and the assessment of its impact on the development of toxic megacolon in patients with CDI. Results without statistical significance. For quantitative parameters, patients were compared using the Mann–Whitney U test. The Chi-square test and Fisher’s exact test were used to compare qualitative parameters.

	All Cohort		Without TM		With TM		*p*-Value
	No.:	%	No.:	%	No.:	%	
Number	31		22	71.0%	9	29.0%	-
Gender							0.106
male	20	64.5%	12	54.5%	8	88.9%	
female	11	35.5%	10	45.5%	1	11.1%	
Age over 65	15	48.4%	10	45.5%	5	55.6%	0.704
BMI over 30	17	54.8%	14	63.6%	3	33.3%	0.233
Hypertension	15	48.4%	9	40.9%	6	66.7%	0.252
Cardiac disease	8	25.8%	5	22.7%	3	33.3%	0.660
DM	7	22.6%	4	18.2%	3	33.3%	0.384
CRF	4	12.9%	3	13.6%	1	11.1%	1
CVI	2	6.5%	1	4.5%	1	11.1%	0.503
Dialysis	1	3.2%	1	4.5%	0	0.0%	1
Cancer	1	3.2%	0	0.0%	1	11.1%	0.290
Bronchial asthma	3	9.7%	2	9.1%	1	11.1%	1
Pregnancy	1	9.1%	1	10.0%	0	0.0%	1
Thyroid disease	5	16.1%	3	13.6%	2	22.2%	0.613
COPD	1	3.2%	0	0.0%	1	11.1%	0.290
ARDS	24	77.4%	18	81.8%	6	66.7%	0.384
Septic state	24	77.4%	16	72.7%	8	88.9%	0.639
Mechanical ventilation	23	74.2%	18	81.8%	5	55.6%	0.185
Corticosteroid treatment—dexamethasone	29	93.5%	20	90.9%	9	100.0%	1
ABT treatment before ICU hospitalization							0.353
none	3	10.3%	3	15.0%	0	0.0%	
cephalosporin	18	62.1%	11	55.0%	7	77.8%	
carbapenem	4	13.8%	4	20.0%	0	0.0%	
penicillin	5	17.2%	3	15.0%	2	22.2%	
others	1	3.4%	1	5.0%	0	0%	
ABT treatment in ICU							
cephalosporin	18	64.3%	11	57.9%	7	77.8%	0.417
carbapenem	4	13.8%	4	20.0%	0	0.0%	0.280
penicillin	5	17.2%	3	15.0%	2	22.2%	0.633

ABT—antibiotic therapy, ARDS—acute respiratory distress syndrome, BMI—body mass index, COPD—chronic obstructive pulmonary disease, CRF—chronic renal failure, CVI—chronic venous insufficiency, DM—diabetes mellitus, ECMO—extracorporeal membrane oxygenation, ICU—intensive care unit, TM—toxic megacolon.

**Table 2:** In the initial results, laboratory values of nutritional status, liver function, inflammation, blood count, coagulation, and basic ions, and the relationship to the development of toxic megacolon, we see significantly higher initial CRP values. In brackets is the range of physiological values. For quantitative parameters, patients were compared using the Mann–Whitney U test.

		All Cohort	Without TM	With TM	*p*-Value
Albumin (32–48 g/L)	Median	33.8	32.1	34.0	0.571
	Minimum	22.0	22.0	27.7	
	Maximum	55.8	55.8	37.0	
Creatinine (64–104 umol/L)	Median	82.0	87.0	77.0	0.384
	Minimum	45.0	45.0	54.0	
	Maximum	296	296	114	
CRP (0–10 mg/L)	Median	126	107	237	0.037
	Minimum	5	6	5	
	Maximum	465	308	465	
Leukocytes (4–10^9^ L)	Median	9.39	9.43	9.05	0.514
	Minimum	3.91	3.91	4.50	
	Maximum	36.1	36.1	22.7	
Procalcitonin (0–5 ug/L)	Median	0.29	0.31	0.14	0.383
	Minimum	0.06	0.06	0.08	
	Maximum	3.99	3.99	2.18	
IL 6 (2.7–50 ng/L)	Median	37.4	39.5	35.2	0.979
	Minimum	1.5	2.2	1.5	
	Maximum	50,000	50,000	259	
Hematocrit (0.4–0.5)	Median	0.40	0.40	0.41	0.445
	Minimum	0.30	0.30	0.30	
	Maximum	36.0	0.4	36.0	
Thrombocites (150–400^9^ L)	Median	225	228	223	0.845
	Minimum	118	118	160	
	Maximum	487	487	388	
D-dimer (0–750 ug/L)	Median	1487	2165	811	0.081
	Minimum	348	348	446	
	Maximum	254,170	254,170	133,230	
Neutrophils (45–70%)	Median	85.3	85.4	82.0	0.728
	Minimum	51.9	51.9	61.6	
	Maximum	92.9	91.6	92.9	
Antithrombin III (80–120%)	Median	87.0	89.5	65.0	0.103
	Minimum	60.0	64.0	60.0	
	Maximum	117.0	117.0	112.0	
Heparin/lmwh (0.0–0.05 IU/L)	Median	0.25	0.26	0.25	0.604
	Minimum	0.03	0.04	0.03	
	Maximum	0.78	0.78	0.39	
Potassium (3.5–5.1 mmol/L)	Median	4.14	4.17	4.14	0.845
	Minimum	3.28	3.28	3.59	
	Maximum	5.19	5.17	5.19	
Phosphorus (0.78–1.65 mmol/L)	Median	1.09	1.10	1.03	0.213
	Minimum	0.32	0.32	0.57	
	Maximum	2.33	2.33	1.25	
Calcium (2.18–2.6 mmol/L)	Median	2.02	1.99	2.06	0.060
	Minimum	1.73	1.73	1.96	
	Maximum	2.37	2.25	2.37	

CRP—C reactive protein, IL-6—interleukin 6, LMWH—low-molecular-weight heparin.

**Table 3:** The influence of primary/self-infection and nosocomial infection acquired during ICU hospitalization on the development of TM.

	All Cohort		Without TM		With TM		*p*-Value
	No.:	%	No.:	%	No.:	%	
Primary infection	11	35.5%	8	36.4%	3	33.3%	1
Klebsiella species	24						0.077
Klebsiella pneumoniae ESBL+	21	67.7%	13	59.1%	8	88.9%	0.205
Klebsiella variicola ESBL+	3	9.7%	2	9.1%	1	11.1%	1
Enterobacteriaceae	3	9.7%	3	13.6%	0	0.0%	0.537
Staphylococcus haemolyticus	5	16.1%	3	13.6%	2	22.2%	0.613
Burkholderia multivorans	8	25.8%	5	22.7%	3	33.3%	0.660
species Candida	6	19.4%	4	18.2%	2	22.2%	1
Serratia marcescens ESBL+	6	19.4%	6	27.3%	0	0.0%	0.145
Enterococcus faecium	8	25.8%	7	31.8%	1	11.1%	0.379
Pseudomonas aeruginosa	9	29.0%	6	27.3%	3	33.3%	1
NI—other	6	19.4%	5	22.7%	1	11.1%	0.642

ESBL—extended spectrum beta-lactamase, NI—nosocomial infection, TM—toxic megacolon primary infection: Candida albicans 5×, herpes simplex 2×, Staphylococcus aureus 2×, Mycoplasma pneumonie 1×, Enterococcus faecium 1×; NI—other: Morganella morganii, Proteus mirabilis, Aspergillus fumigatus, Micrococcus luteus, Stenotrophomonas maltophilia, Acinetobacter baumannii.

**Table 4:** Significant predictors of survival from a positive PCR test. Parameters with the relative risk (RR) > 1 increase the risk of exitus. Parameters with RR < 1 decrease the risk of exitus. RR corresponds to the change in the risk of exitus with a unit change in the parameter value. Significant parameters increasing the risk of exiting: age, presence of chronic venous insufficiency, cardiac disease, mechanical ventilation, presence of Candida. Significant parameters that reduce the risk of exitus include corticosteroid treatment, ABT prophylaxis with cephalosporine, and current Klebsiella infection.

	*p*-Value	RR	95.0% CI for RR	
			Lower	Upper
Gender	0.553	0.729	0.256	2.071
Age	0.042	1.034	1.001	1.069
Age over 65	0.206	1.867	0.709	4.918
BMI	0.594	0.980	0.909	1.056
BMI over 30	0.340	0.629	0.242	1.632
Time from PCR test positivity to admission to the ICU (days)	0.524	0.969	0.880	1.067
Hypertension	0.514	1.375	0.529	3.574
Cardiac disease	0.021	3.220	1.194	8.680
DM	0.789	1.166	0.379	3.583
CRF	0.410	1.693	0.484	5.914
CVI	0.021	7.445	1.352	41.0
Dialysis	0.119	5.516	0.644	47.2
Cancer	0.081	7.037	0.786	63.0
Bronchial asthma	0.741	1.283	0.293	5.622
Thyroid disease	0.658	0.716	0.164	3.136
COPD	0.554	0.046	0.000	1219
ARDS	0.074	6.341	0.838	48.0
Septic state	0.182	2.737	0.625	12.0
Mechanical ventilation	0.045	7.909	1.044	59.9
ECMO	0.821	1.186	0.269	5.220
Remdesivir therapy	0.539	0.629	0.144	2.755
Corticosteroid treatment—dexamethasone	0.011	0.124	0.025	0.621
Inosine pranobex therapy	0.398	0.043	0.000	64.1
ABT cephalosporine	0.029	0.330	0.122	0.890
ABT carbapenem	0.191	2.321	0.657	8.198
ATB penicillin	0.213	2.051	0.663	6.348
Albumin	0.226	0.941	0.853	1.038
Creatinine	0.514	1.002	0.995	1.009
Initial CRP	0.567	0.999	0.994	1.003
Leukocytes	0.544	0.976	0.903	1.055
Procalcitonin	0.544	0.859	0.526	1.403
IL 6	0.353	1.001	0.999	1.002
Sodium	0.336	1.665	0.589	4.708
Potassium	0.776	0.839	0.252	2.799
Calcium	0.193	0.098	0.003	3.245
Hematocrit	0.079	1.057	0.994	1.123
Thrombocites	0.252	0.996	0.990	1.003
D-dimer	0.603	1.000	1.000	1.000
Neutrophils	0.859	1.005	0.949	1.065
Antithrombin III	0.737	1.005	0.976	1.035
Heparin/lmwh	0.709	1.728	0.098	30.5
CDI—toxin	0.441	0.687	0.265	1.785
Primary infection0/1	0.932	0.958	0.354	2.595
Klebsiella species	0.031	0.341	0.128	0.904
Klebsiella pneumoniae ESBL	0.106	0.449	0.170	1.186
Klebsiella varicola ESBL	0.296	0.039	0.000	17.0
Enterobacter	0.492	0.493	0.065	3.717
Staphylococcus haemolyticus	0.390	0.523	0.119	2.292
Burkholderia multivorans	0.773	1.166	0.410	3.315
Candida	0.039	3.091	1.059	9.024
Serratia marcescens ESBL+	0.647	0.747	0.214	2.605
Enterococcus faecium	0.642	1.281	0.451	3.639
Pseudomonas aeruginosa	0.150	2.046	0.773	5.417
NI other	0.106	0.188	0.025	1.422
Toxic megacolon	0.794	1.150	0.404	3.271
No. of operations: 1	0.385	1.570	0.567	4.344
No. of operations: 2	0.902	0.879	0.112	6.883
Clinical frailty scale	0.504	1.198	0.705	2.038
Initial ATLAS score	0.189	1.212	0.910	1.615
ATLAS score with CDI	0.090	1.259	0.965	1.643

ARDS—acute respiratory distress syndrome, BMI—body mass index, COPD—chronic obstructive pulmonary disease, CRF—chronic renal failure, CVI—chronic venous insufficiency, DM—diabetes mellitus, ECMO—extracorporeal membrane oxygenation, ICU—intensive care unit, TM—toxic megacolon.

## Data Availability

The data that support the findings of this study are available in Open Science Framework https://osf.io/exscv/?view_only=70ef395f540a4e0790e4828918e53798, accesed on 13 June 2022.

## Abbreviations

ACE2	angiotensin-convertase enzyme 2
BMI	body mass index
CDI	Clostridium difficile infection
COVID-19	coronavirus disease 2019
ESR	erythrocyte sedimentation rate
NO	nitric oxide
SARS-CoV-2	severe acute respiratory syndrome coronavirus 2
TM	toxic megacolon
TMPRSS2	transmembrane protease serine 2
UC	ulcerative colitis
WHO	World Health Organization
